# Impact of Probiotic Bacteria on Respiratory Allergy Disorders

**DOI:** 10.3389/fmicb.2021.688137

**Published:** 2021-06-21

**Authors:** Dominika Jakubczyk, Sabina Górska

**Affiliations:** Laboratory of Microbiome Immunobiology, Hirszfeld Institute of Immunology and Experimental Therapy, Polish Academy of Sciences, Wrocław, Poland

**Keywords:** probiotic, respiratory allergy, oral supplementation of probiotics, intranasal supplementation of probiotics, asthma, rhinitis

## Abstract

Respiratory allergy is a common disease with an increased prevalence worldwide. The effective remedy is still unknown, and a new therapeutic approach is highly desirable. The review elaborates the influence of probiotic bacteria on respiratory allergy prevention and treatment with particular emphasis on the impact of the current methods of their administration – oral and intranasal. The background of the respiratory allergy is complex thus, we focused on the usefulness of probiotics in the alleviation of different allergy factors, in particular involved in pathomechanism, local hypersensitive evidence and the importance of epithelial barrier. In this review, we have shown that (1) probiotic strains may vary in modulatory potential in respiratory allergy, (2) probiotic bacteria are beneficial in oral and intranasal administration, (3) recombinant probiotic bacteria can modulate the course of respiratory allergy.

## Introduction

The hypersensitivity also called an allergy, is one of the most underestimated and marginalized diseases. According to the European Academy of Allergy and Clinical Immunology (EAACI), around 40% of people suffer from allergy rhinitis worldwide, and asthma affects around 334 million people globally (Agache et al., 2019). Allergic diseases can be misdiagnosed by doctors, resulting in inappropriate treatment (Heffler et al., 2015; Aljfout et al., 2016; Ulrich et al., 2017). On the other hand, a significant percentage of patients with rhinitis symptoms try to self-medicate and select their drugs in a pharmacy shop based on the marketing advertisements, family/friend suggestion and others (Tan et al., 2017). The costs related to health care and temporary disability due to allergic diseases are increasing significantly worldwide (Dierick et al., 2020). The term ‘respiratory allergy’ generally describes the group of symptoms appearing after inhaling allergens. Respiratory allergy diseases can be divided by their placement within the upper and lower airways, but chronic contact with allergens can be demonstrated by eye or ear symptoms as well (Kreiner-Møller et al., 2012; Michailopoulos et al., 2017). Respiratory allergies can display different forms and symptoms. They can be triggered by an allergen such as pollen, house dust mites, animal dander, molds, and inhaled food proteins (Jeebhay et al., 2019; Eguiluz-Gracia et al., 2020). The most common respiratory disorders, widely linked with hypersensitivity type I, are rhinitis and asthma. Both of those terms describe general clinical symptoms. Nevertheless, the background of rhinitis and asthma are strongly diversified (Bhargava et al., 2011; Baos et al., 2018). Rhinitis is a general term describing the inflammation of nasal mucosa and typically pronounced by a runny nose, nasal itching, and sneezing. However, it can have different causes. In clinical practice, rhinitis can be related to viral infection (called infectious rhinitis) (Hellings et al., 2017). It can be trigger by irritants, drugs, hormonal imbalance, pregnancy, age, and others or have the idiopathic character, which is generally called the non-allergic, non-infectious rhinitis) (Hellings et al., 2017; Mullol et al., 2020). Rhinitis caused by allergens and mediated by IgE is called allergic rhinitis (AR) (Passali et al., 2018; Jungewelter et al., 2020). Additionally, some of the anatomical abnormalities, presence of foreign bodies in the upper airways, nasal polyps, primary ciliary dyskinesia can be manifested in the same way, therefore rhinitis demand a careful diagnosis (Hellings et al., 2017; Dykewicz et al., 2020). Similarly to rhinitis, asthma is an umbrella term. According to World Health Organization (WHO) asthma is a chronic inflammation of the air passages in the lungs, which is reflected in oversensitivity to irritants^1^. The narrowed bronchial tubes and excess of mucus production lead to the reduction of the airflow. This disease is manifested by wheezing, chest tightness, or paroxysmal cough. The conventional asthma division is based on its background, allergic or non-allergic. In contrast to the non-allergic form, allergic asthma usually has an early onset, it is triggered by allergens and mediated by the IgE. However, in clinical practice, this basic classification is not sufficient. Determination of the exact type of asthma enables personalized medicine and better therapeutic outcomes (Agache, 2019; Kuruvilla et al., 2019). Recently, the endotypes and phenotypes of asthma have been distinguished. The term ‘endotype’ refers to the mechanism of action, ‘phenotype’ includes the external manifestation of asthma (Agache, 2019). The main endotypes are (1) Th2-high (eosinophilic form, with an increase in IgE, FeNO, IL-13, IL-5), and (2) Th2-low (non-eosinophilic, neutrophilic or paucigranulocytic form, with an increase in IL-6, myeloperoxidase). The Th2 predominant type includes atopic asthma, late-onset form and aspirin-exacerbated respiratory disease. The Th2-low form include phenotypes with non-atopic background, related to smokers, to obesity and age (Kuruvilla et al., 2019). Moreover, the recent studies underline role of IL-17 in asthma. The endotype Th2/Th17-high is characterized by an increase especially in IL-4, IL-17, IL-1β, IL-6, IL-23, complement protein (C3a), serum amyloid A and p38 MAPK. Whereas, the increased level of myeloperoxidase, IL-8, IL-1α, IL-6, C3a, G-CSF, GM-CSF is observed in the endotype Th2/Th17-low. While IL-8 derived from airway cells seems to regulate the cells infiltration, its presence is linked with the neutrophilic form, whereas its absence is linked with the paucigranulocytic’s Th2/Th17-low phenotype (Liu et al., 2017; Agache, 2019).

The allergic respiratory symptoms can occur together (e.g., rhinitis and asthma), but the direct link between them was not proved (Bergeron and Hamid, 2005; Fokkens and Braunstahl, 2005). Moreover, allergic respiratory diseases commonly coexist, not only within the respiratory tract (rhinitis and asthma) but also with a food allergy or atopic dermatitis (Roduit et al., 2017). The specific clinical approach is widely underlined in the available literature and the accessible data on respiratory allergy mark that all symptoms should be evaluated ‘globally,’ not only according to the affected place and gate of allergen access (Passali et al., 2018).

The positive effect of probiotic for disease symptoms alleviation is noted in many diseases, especially within the gastrointestinal tract. However, the probiotic utility in respiratory allergy is a new trend. Even though the exact relationship between bacteria and the atopic host has not been described yet, the clinical and biomedical evidence for the positive effect of probiotics on the allergy has been reported. The review presented focuses on the application of probiotics in respiratory allergy. The authors discuss the oral and the intranasal administration of probiotics in different experimental models as well as biomedical trials. Additionally, the authors systematize the knowledge on respiratory allergy, including the mechanisms, clinical phases, and the importance of the epithelial barrier.

## The Respiratory Tract as Protection From Foreign Antigens

### The Physiological Condition

A respiratory tract is a powerful tool for contact with the environment and as a defense against harmful organisms and substances. Depending on the measurement method, it is accepted that the general size of the lung is around 100 m^2^, the nose with the sinuses provides even 200 cm^2^, and only the alveolar surface is 50 times bigger than the skin barrier (Fröhlich et al., 2016; Yuksel and Turkeli, 2017). The amount of inhaled air comes to 600 L daily. Each day, in a single breath, millions of different particles are inhaled and distributed through the respiratory tract. Some of them are removed, some are deposited. The deposition of these particles depends on many factors, such as their size, shape, surface properties, etc. (Heyder, 2004). From an anatomical aspect, the airways are built of the upper and lower respiratory tracts. The upper part consists of the nose, sinuses, pharynx and larynx, the lower part, of the trachea, bronchi, bronchioles and alveoli. Within the airways, the lymphatic tissues are well developed. Human NALT (nose- associated lymphoid tissue) is a part of the mucosa-associated lymphoid tissue and consists of disseminated structures of lymphoid tissue. It contains T cells, B cells, dendritic cells, macrophages. It was found in the child population in different locations, including the upper nasal cavity, the middle concha, inferior nasal concha, superior, nasal concha, sinus, palate, and others. NALT among an adult’s population was not described (Pabst, 2015). BALT (bronchus-associated lymphoid tissue) is also part of the mucosa-associated lymphoid tissue and it is present within the bronchial mucosa. The presence of BALT is found in the children and young adults’ pmostly (Heier et al., 2011). Generally, the occurrence of the BALT and NALT vary among the species (human, rat, mouse, etc.), but they are important for immune response and tolerance induction (antigen uptake, presentation, and T and B cells activation), especially during childhood. Their main function is to provide a specific immunological response leading to immunoglobulin, especially IgA production (Pilette et al., 2001; Kato et al., 2013). The epithelial cells are endowed with pattern recognition receptors (PRRs), such as toll-like receptors 1-10 (TLR1-10), a retinoic acid-inducible gene I (RIG-I)-like receptors (RLRs), dectin, damage-associated molecular pattern (DAMP), and proteases activated receptors, which allow active identification of microbial, viral or allergic antigens (Willart and Hammad, 2010). The unspecific response is based on the secretion ability of the epithelial cells and their phagocytotic ability (Juncadella et al., 2013). The epithelium contains three main sorts of cells: ciliated, secretory, and basal (Tata and Rajagopal, 2017). The ciliated cells are responsible for the mechanical removal of foreign antigens. The secretory cells produce mucus, antimicrobial factors as well as they can neutralize toxins. The basal cells have a progenitor function (Hiemstra et al., 2015; Tata and Rajagopal, 2017). The epithelial cells provide a mechanical barrier due to tight connections between single cells. These connections are created by secreted mucus as well as produced proteins, such as occludin and claudin (tight junctions, localized in apical parts), adherent junctions (localized in lateral membranes), and junctional adhesion molecules (Brune et al., 2015; Toppila-Salmi et al., 2015). In the neighborhood of the basal cells and the basement membrane, cells such as granulocytes, lymphocytes, macrophages, dendritic cells, and mast cells are located, which results in permanent contact between the external and the internal environments (Bourdin et al., 2009; Amin, 2015; Smith et al., 2019). The respiratory tract surface must face millions of environmental factors every second, and it must recognize which agents are harmful. Therefore, the presence of highly specialized cells is crucial. This allows for permanent sampling and agent recognition. The key players are the dendritic cells, which are equipped with pathogen-associated and damage-associated molecular patterns, allowing the detection of foreign agents. Dendritic cells can locate allergens (Humeniuk et al., 2017; Morianos and Semitekolou, 2020). In a physiological state, the allergens are neutral and do not activate any further cascade. Nevertheless, in a predisposed organism or during chronic exposure to the allergen, the breakdown of the respiratory barrier take place. This leads to the activation and maturation of the cells and further immunological activity (Willart and Hammad, 2010).

### The Breakdown of the Respiratory Barrier

The breakdown of the respiratory tract barrier results in sensitivity. The unsealing of the border has both a mechanical and a functional character (Mattila et al., 2011). The functional one comes down to the loss or diminution of mucus production, antimicrobial factors, etc. The mechanical lesion relies on the loss of a tight connection between the cells. The indirect path of mechanical dysfunction is grounded in pro-inflammatory mediators and can be shaped by the allergens as well. For instance, *Phleum pratense* (Timothy grass) induces chemokine production, thus promoting inflammation and other reactions (Blume et al., 2013). Some air allergens can change the consistency of the epithelial barrier in a direct way. Respiratory allergens can act in a proteases-dependent manner, for instance, some of *Dermatophagoides pteronyssinus* epitopes display enzymatic activity, which directly increases the permeability of the epithelium barrier, decreasing the numbers of tight junctions, e.g., occludin, claudin JAMA, zonula occluded-1 (Wan et al., 1999, 2000; Matsumura, 2012; Henriquez et al., 2013), pollen extracts like Giant Ragweed (*Ambrosia trifida*), White Birch (*Betula pendula*) or Kentucky Blue Grass decrease occludin and claudin occurrence (Runswick et al., 2007; Georas and Rezaee, 2014). On the contrary, some allergens do not display enzymatic activity and work in a proteases-independent manner. This group of allergens has some structures which directly activate the receptors on the cells, for instance, cockroach scat can directly affect neutrophils by a TLR-2 activation (Traidl-Hoffmann et al., 2005), and *D. pteronyssinus* (Der p 7) contains bactericidal permeability-increasing like protein which directly modulate the immunological system (Mueller et al., 2010). Additionally, respiratory virus infections, cigarette smoke, air pollution, toxins, which are inhaled every minute, can disturb the barrier directly as well (Georas et al., 2010; Georas and Rezaee, 2014). Besides the above external factors, the internal environment of the organism, such as its genetic aspect, or accompanying other diseases/inflammatory states, can also diminish the function of the respiratory barrier.

## Sensitization. Hypersensitivity/Allergic Reactions

The term ‘allergy’ was coined by Clemens von Pirquet in 1906 to describe the changes in the reactivity of the organism both in a hypersensitive and a hyposensitive manner after contact with substances called allergens (Huber, 2006). The author’s most important conclusion was that reaction depends not only on exogenous stimulation but also on the specific endogenous processes (Huber, 2006). The first classification of a hypersensitive reaction was introduced by Gell and Coombs in 1963. This elaboration described I–IV forms of hypersensitivity based on the immunological system’s involvement. Although it is more than half a century since the publication of this paper, the main assumptions of this categorization are still valid and are the basis of modern immunology (Igea, 2013; Marshall et al., 2018). Currently, hypersensitivity and allergy are used interchangeably. For the needs of this article, the authors will focus on the first type of hypersensitivity, according to Gell and Coombs.

### Sensitization

The first contact with the allergen for an allergy-predisposed person leads to IgE production. The allergen can enter the airway by the active damage to the epithelium or penetrate through the previously damaged barrier. The identification of the allergen lies in receptor recognition by immunological and non-immunological components. Epithelial cells are equipped with the receptors such as PPR (pattern recognition receptors) and PAR (protease-activated receptor). Their activation results in cytokines release and further activation of immunological cells. Simultaneously, the allergen is tracked down by the ‘antigen-presenting cells’ (APC), which take it up and process. Dendritic cells which perpetually sample the external and internal stimuli are especially important. They are equipped with the receptors, including C-type lectin receptors [mannose receptor (MP), dendritic cell-specific intercellular adhesion molecule 3-grabbing non-integrin (DC-SIGN)], TLR, FcεRI, which enable allergen tracking and further processing (Salazar and Ghaemmaghami, 2013). The first step involves allergen uptake, its degradation and generation of the phagolysosome. At the same time, the MHC II transfer (from the endoplasmic reticulum to the Golgi apparatus) takes place. The fusion of phagolysosome (containing fragments of allergen) with vesicles (containing the MHC class II) leads to class I-associated invariant chain peptide (CLIP) formation and release of the antigenic peptide on the cell surface (Humeniuk et al., 2017). The antigen presentation usually takes place in the lymph node region. Capturing of the allergens leads to the expression of the specific co-stimulatory molecules (e.g., CD80 and CD86), which affect the T cells. The interaction between APC and T cells is mediated through MHC II with a processed allergen and TCR (T cells receptor) respectively. Generally, under healthy conditions, this contact between APC and T cells leads to a switch of the T cells into Th1 (Galli et al., 2008; Galli and Tsai, 2013), which also stimulate lymphocytes B to produce IgG antibodies. The IgG and especially human IgG4 isotype, show in this case a kind of protective effect in respect of allergies. They can destroy the allergen, block the basophile activation, and block the IgE-binding receptors (Cady et al., 2010; Flicker et al., 2013). If organisms exhibit an allergic tendency, the T cells are switched into a Th2 subpopulation, which has a strong pro-inflammatory role. Formation of the Th2 subpopulation from the Th0 is possible especially through IL-4, IL-13, IL-25, IL-33, TSLP (thymic stromal lymphopoietin), co-stimulation by CD28 (Curotto de Lafaille, 2010). Those cytokines have multiple sources in the organism, including damage epithelial barrier or basophils, which are activated directly by external proteases (Galli et al., 2008; Curotto de Lafaille, 2010). There is also an alternative way of Th2 activation. Recently described group 2 innate lymphoid cells (ILC2s), seem to play a pivotal role in allergic inflammation. ILC2s respond to the cytokines released from damaged epithelial cells (IL-25, IL-33, and TSL), which leads to further cytokines production: IL-4 (impact on Th2 skewing, IgE production by B cells), IL-5 (eosinophils requirement and activation, B activation cells), IL-9 (basophils maturation, mucus production), IL-13 (B cells activation, support dendritic cells in migration to the lymphoid nods, airway remodeling and contraction). Moreover, ILC2s can introduce the processed antigen directly to the T cells, via MHC II molecules (Martinez-Gonzalez et al., 2015; Cheng et al., 2018; Helfrich et al., 2019; Pasha et al., 2019). Induced Th2 cells (by APC and/or ILC2s) stimulate lymphocytes B to produce IgE (switching of antibody classes) (Van Ree et al., 2014). The IgE antibodies connect the mast cells and basophils via receptor FcεRI with high affinity, creating a strong connection. This receptor is also available on the surface of dendritic cells, macrophages, Langerhans cells, eosinophils, and monocytes (Yen et al., 2010; Shin and Greer, 2015). Structurally, the FcεRI receptors consist of α, β, and γγ chains. The α chain is responsible for the IgE anchor. The remaining chains take part in the stabilization of the whole structure, as well as forwarding the signal to the cell. The receptor FcεRII for IgE, and especially in its dissolved form, does not create a direct connection with IgE but seems to have a regulatory purpose (MacGlashan, 2009; Galli and Tsai, 2013). Most of the specific IgE produced are related to the mast cells and are stored among the effector tissue until the next contact with the same allergen. The minor pool of unbound specific IgE circulates freely in the peripheral blood. This readiness is called sensitization, which is necessary for the further development of hypersensitivity type I reaction.

### Hypersensitivity Type I (Allergy)

Hypersensitivity type I (allergy) is an IgE mediated reaction that occurs after a second contact with an allergen. During the second contact with the allergen, the mast cells recognize the allergen via the IgE bound to their surface. This is the first phase in hypersensitivity/allergic reaction development. Mast cells, with the previously anchored IgE, connect the allergen particles. The allergen must be connected by at least two IgE on the mast cell. During the rest/anticipation phase, the FcεRI receptors are evenly located on the cell surface. Linking with the allergen leads to cluster formations, which further lead to cell activation and its degranulation (Kawakami and Blank, 2016). This process starts with the reaction of the gamma and beta chains with a protein kinase, e.g., Syk (spleen tyrosine kinase), Fer (tyrosine-protein kinase), Btk (Bruton’s tyrosine kinase), which causes the activation of two signaling paths – kinase MAP (Mitogen-activated protein) and phospholipase C. The phospholipase C changes the PIPT2 (Phosphatidylinositol 4,5-biphosphate) into IP3 (inositol-1,4,5-triphosphate) and DAG (diacylglycerol). The IP3 is responsible for opening the calcium canal, the DAG for the protein kinase C activation (PKC), which further influences the protein phosphorylation. Both PKC and calcium influence the cytoskeletal shape. The granularity gets closer to the cell membrane and can be released outside. The MAP kinase activation leads to protein phosphorylation processes, which result in leukotriene and prostaglandin arising. The MAP kinase modifies the transcription factors as well (Steelman et al., 2015; Draber et al., 2016; Moeller et al., 2019).

The mast cells and basophils produce many factors affecting the pro-inflammatory and allergy processes. These mediators can be divided into three groups: (1) those stored in the granularity (preformed), (2) mediators produced *de novo*, and (3) cytokines. Histamine is located among mediators stored in the granularity and can be released from cells by an immunological (IgE) stimulus, as well as in a non-immunological way. Histamine, reacting through the receptors H1 or H2 on the effector cells, is responsible for the local pro-inflammatory reactions, increasing vascular permeability and a smooth muscle spasm. Histamine is also a strong chemoattractant and promotes prostaglandin secretion. During very intense degranulation of the mast cells, histamine can penetrate to the blood and presents systemic symptoms (anaphylaxis) (Borriello et al., 2017; Thangam et al., 2018). In addition to the histamine, in granularity, the proteoglycans, serine proteases, TNF-α, chemotactic factors are stored.

**FIGURE 1:**
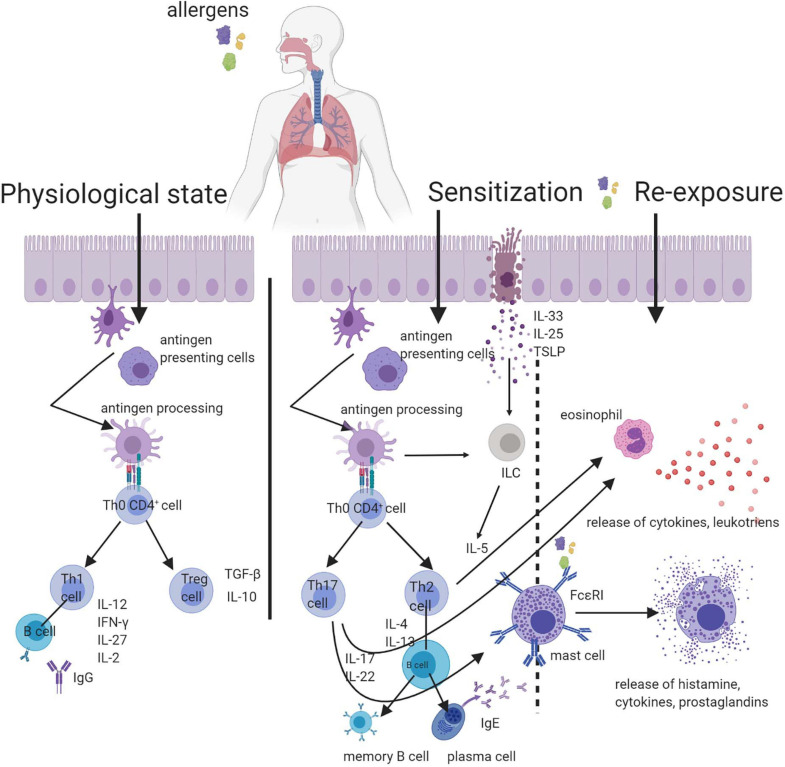
Scheme of respiratory allergy with the inclusion of particles destroying the respiratory barrier. In a physiological condition, the contact with the allergen is not harmful. In the predisposed organism, the first contact with the allergen leads to sensitization. The re-exposure to allergen mobilizes the effector cells and results in the successive stages of an allergic reaction.

Among the mediators produced *de novo* are the metabolites of arachidonic acid [prostaglandins, leukotrienes, PAF (platelet-activating factor), sphingosine phosphorane]. Among cytokines produced by the mast cells, the most important are IL-4, IL-1, IL-3, IL-10, IL-13, TNF-α, TNF-β (Moon et al., 2014). The mast cells can be activated, not only by the allergen and IgE, but also by the complementary proteins (C3a, C4a, and C5a), cytokines (IL-4, IL-5, and IL-8), activators of TLR, acetylcholine, methacholine, temperature, stress, UV radiation, food addition such as preservatives coloring and others (Deacock, 2008; da Silva et al., 2014; Wang et al., 2016; Caslin et al., 2018). This unspecific activation of mast cells clinically can cause similar symptoms to the allergy type I but without the IgE involvement, which can lead to an incorrect diagnosis (Cardet et al., 2013; González de Olano et al., 2016). Clinically the whole process of hypersensitivity reaction type I has two stages. The clinical manifestations are reliant on the place where cell degranulation occurred and can have an organ-specific or systemic character. The intensity and nature of this chamfer depend on many factors, including the numbers of IgE and mast cells, the type of allergen, its pathway, and level of sensitization. The pro-inflammatory mediators (e.g., histamine) released from the mast cell have chemotactic and vasodilatory importance and increase the permeability of the blood vessels, which is a cause of inflammatory infiltration, oedema, lacrimation, mucus increase. The innervation of the neuronal fiber triggers bronchospasm, dyspnoea, itchiness, redness (Joos et al., 2000; Hansen et al., 2004; Galli et al., 2008). The early phase starts just after a few seconds/minutes after contact with an allergen and takes 1–2 h. For the late phase of the reaction, the characteristic is a contribution of basophils, eosinophils, lymphocytes T and pro-inflammatory mediators produced *de novo*. The late phase is not so intensive as the early phase but lasts longer (Galli et al., 2008). The general scheme of respiratory allergy is introduced in Figure 1.

### Allergy-Predisposing Factors

Since generally allergy has a multi-faceted matrix, there are many factors that can increase the likelihood of its development. The stimuli are divided according to the environment where they come from. Essentially, genetic background belongs to the internal factors. The inheritance of proclivities to allergy is called atopy (that term describes the strong tendency to IgE production as well) (Amin, 2015). Nevertheless, the genetic impact was first described in 1916, and current sources confirm its importance in the development of respiratory disorders (Cooke and Vander, 1916; Ortiz and Barnes, 2015; Portelli et al., 2015; Loxham and Davies, 2017). The frequency of family burden depends on the disease, for asthma (generally) is estimated around 35–95%, for allergic rhinitis 33–91%, 30–66% bronchial hyperresponsiveness (Ober and Yao, 2011). A powerful external factor stimulating an allergy occurrence is air pollution. Automobile air contamination influences the organism in a few ways. It can stimulate the local IgE production, without influencing other subtypes of immunoglobulins, which affects Th2 skewing, increasing the production of IL-4, IL-5, IL-6, and IL-10 (Lee et al., 2013), accelerating dendritic cell maturation (Matthews et al., 2016), directly disturbing the tight junctions within the respiratory tract (Wang et al., 2012). Air pollution impacts the mucosal immunity in the respiratory tract by activation the redox reaction and increases oxidative stress (Huff et al., 2019). The pollutants affect asthmatic individuals in a few ways. Their high concentration directly triggers the irritant and inflammation on the airway neuroreceptors and epithelium, a lower concentration of ozone and/or nitrogen dioxide induce inflammation and hyperresponsiveness via impact on the genes [airway responsiveness genes, antioxidant genes, and immune response genes (Guarnieri and Balmes, 2014)]. Among the environmental stimuli, one of the most important seems to be the contact with foreign antigens. This issue is multifaceted. The ‘hygenic theory’ was introduced in 1989 and was based on the observation, that ‘allergy is a post-industrial epidemy’ (Strachan, 1989). Because of the low exposure to allergens (excessive hygiene, less exposure to the world of plants and animals), the organism cannot learn and acquire tolerance, which results in a change of balance toward Th2 and a decrease in the body’s resistance to foreign antigens. The number of allergies is lower in areas where the diversity of nature is higher (Haahtela, 2009; Okada et al., 2010; Ege et al., 2011). Recently, the connection between parasitic infections and allergy was noted (Cruz et al., 2017; Wu et al., 2017). Although the Th2 response is predominant during chronic helminth infections, allergic symptoms are decreased, which could be an explanation for a lower allergy occurrence in developing countries (Smits et al., 2010; Stein et al., 2016). However, acute helminth infections do not confirm this phenomenon (Smits et al., 2010). It was demonstrated as well that the measles virus keratinocytes infection can protect against atopic dermatitis by changing the cytokine profiles, which are important in the allergy mechanism (e.g., in CCL26 and TSLP) (Gourru-Lesimple et al., 2017). Mycobacterium tuberculosis chronic infections can protect from asthma and allergic rhinitis, probably by the increase of the Th1 response among children living in a tuberculosis-endemic area (Obihara et al., 2006). On the other hand, scientific publications indicate that some of the viral stimuli affect allergy development, e.g., the Respiratory Syncytial Virus (RSV) infection in early childhood is one of the factors predisposing to the allergy in adult life, but the mechanisms of this issue are still unclear (Schauer et al., 2002; Edwards et al., 2017).

## Microbiome of the Respiratory Tract

For years, because of the lack of sensitive methods, there was a strong belief that the lower respiratory tract is sterile. In 2007 the National Institute of Health (NIH, United States) started a 5-year international project called ‘Human Microbiome Project’ (HMP) and the aim of this undertaking was the characterization of the microbiome of the gastrointestinal tract, mouth, vagina, and skin (Turnbaugh et al., 2007; Peterson et al., 2009). The separate NIH project (‘Lung HIV Microbiome Project,’ LHMP, period: 2009–2015) was dedicated to the lung’s habitats^2^. Nevertheless independently, Hilty et al. (2010) introduced the first original work, based on 16S rRNA method, which confirmed not only the presence of a lung microbiome but additionally showed the differences in microbiome structure in healthy individuals, asthma and chronic obstructive pulmonary disease (COPD). This has started a new approach with a strong consideration of the human microbiome in the aetiology of respiratory tract diseases. Today it seems to be obvious that airways are an important microbiome habitat that can shape the immunological status of the host. In contrast to the gut microbiome, the lung ‘community’ marks a lower density of bacterial cells [around 20–1,252 bacterial cells per 1,000 human cells (Sze et al., 2012)]. The main group of bacteria characterized within the lower airway tract are *Firmicutes*, *Bacteriodetes*, *Proteobacteria*, *Fusobacteria*, and *Actinobacteria* (Moffatt and Cookson, 2017). The colonization starts immediately after birth. The composition of airway microbiota depends on the maturity of the respiratory system of the newborn (Wypych et al., 2019). Moreover, early colonization by *Corynebacterium* spp., *Dolosigranulum* spp., and *Moraxella* spp., is linked with a healthy respiratory track (Man et al., 2017). The process of bacteria colonization is complex. Bacteria have to attach to the epithelium layer to avoid removing it with inhaled air. This process can be mediated by binding to mucus, association with host carbohydrates or surface proteins. The airway bacteria must use locally available nutrients, which are much poorer than those found in the intestine and have to avoid the host’s immune system response (Siegel and Weiser, 2015). The role of the microbiome in the respiratory tract is not widely studied, especially in comparison with the role of the gut microflora. Nevertheless, it is considered that the main function is protection against pathogen colonization, mutual regulation of the population and maintenance of host homeostasis. Bacteria colonizing the airway form a specific community that regulates itself. For example, *Corynebacterium* spp. and *Staphylococcus* spp. modulate the growth of each other, *Corynebacterium striatum* upregulate the commensal properties of *Staphylococcus aureus*, and additionally *Corynebacterium accolens* (by the metabolism of host triacylglycerols into free fatty acids) block the pneumococcal expansion (Man et al., 2017). Microbiota shapes the mucus production, which modulates the barrier function, remodeling the organ structure (increase the alveolar presence), as well as promote the tolerance of the immune system (Mathieu et al., 2018). The altered microbiota has an impact on respiratory diseases as well. Many studies indicated that patients with allergy, asthma, and chronic obstructive pulmonary disease (COPD) have disturbed the microbiome composition, which can accelerate the further pathological processes (Choi et al., 2014; Huang, 2015; Depner et al., 2017; Leiten et al., 2020). Asthmatic patients differ in the microbiota composition as well, and it seems to be linked with the phenotype of the disease. The special differences are visible between eosinophilic and neutrophilic asthma. The neutrophilic form of asthma is linked with: (1) the increase of the disease severity, (2) the increase of IL-17 level (which promote the neutrophile infiltration), (3) the conventional treatment-refractory (steroids promote the neutrophils survival) (Wang and Wills-Karp, 2011; Ray and Kolls, 2017). Patients with the neutrophile’s domination in the sputum were characterized by the lower diversity and numbers of detectable bacterial taxa in the airway, which might have an impact on the infection susceptibility and response to asthma treatment (Taylor et al., 2018). Microbiota can be an active player in disease development as well. Yadava et al. (2016) reported, that in the murine model of chronic pulmonary inflammation, the microbiome induced the IL-17A production and therefore promoted chronic inflammation. In another murine model, Jin et al. (2019) indicated, that respiratory microbiota induced inflammation and lung adenocarcinoma. Bacteria upregulated Myd88-dependent IL-1β and IL-23 production by myeloid cells, which was associated with Vγ6 + Vδ1 + γδ T cells activation, further production of IL-17 and other factors promoting tumor-genesis.

The direct mechanism of bacteria-host crosstalk is still blurred. However, it is clear, that the respiratory microbiome is a key player in airway health maintenance and disease acceleration. Bacteria composition changes under different condition and seems to rather a part of the ‘big microbiome system’ that local niche of bacteria.

### The Gut-Lung Axis

There is a growing body of evidence that the respiratory tract’s microbiome is associated with the gut microbiome (Marsland et al., 2015; Wedgwood et al., 2020). Generally, species included in the human microbiome are largely similar, but the percentage share varies depending on the habitat (lung and gut). What is more interesting, species domiciled in different habitats can influence each other. Recently, the role of the gut-lung axis has been increasingly emphasized in the available literature (Marsland et al., 2015; Budden et al., 2016; Hauptmann and Schaible, 2016). Clinically it was noted that diseases of the gastrointestinal tract also have a reflection in the respiratory tract. Even half of the patients with inflammatory bowel syndrome also suffer from pulmonary disorders (Yazar et al., 2001), and the structure of the microbiome in a healthy group and patients suffering from lung/gut perturbations, differ from each other (Frati et al., 2019). The direct mechanism of this intercommunication is not clear. One of the theories underlines the importance of the SCFA (short-chain fatty acids) such as acetate or butyrate, which have anti-inflammatory and immunomodulatory properties (Dang and Marsland, 2019). The SCFA produced by intestinal bacteria from food fibers have systematic influence, improve lung immunity, increase the peripheral Treg, Th1 and Th17 subpopulations, modulate cytokine expression and stimulate the progenitor cells (dendritic cells and macrophages) in bone marrow (Dang and Marsland, 2019). Nevertheless, the full understanding of the gut-lung cross-talk mechanism is still not clear.

## Respiratory Allergy and the Oral Supplementation of Probiotics

The common therapeutic strategies used for respiratory allergy are focused on the symptoms and include allergen avoidance, antihistamine, or steroid drugs. That approach helps to alleviate the oppressive afflictions. However, it has some limitations such as side effects and steroid-refractoriness in some groups of patients. Allergen immunotherapy is a method of allergen tolerance induction. Nevertheless, it is a long-lasting treatment of varying effectiveness, limited to the specific group of patients with determined allergic trigger factor (Calderón et al., 2015). The modern biological treatments target interleukin blockade, however, they demand a determination of the exact disease’s endotype (Agache and Akdis, 2019). The influence of oral probiotic administration in allergy disease limitation is more and more often emphasized. According to the definition, probiotics are microorganisms that are introduced to the human body by the digestive system, and in an appropriate dose, have a positive influence on the host (Sanders, 2008). The beneficial influence on the gut as the main place of residence for probiotic microorganisms seems to be natural. The onset of probiotic protection dates from fetal life (Rackaityte et al., 2020) when early colonization takes place. Initially, among newborns, a Th2 skewing is observed, and with further development of the immune system, a Th1/Th2 balance is achieved. A lack of this balance triggers sensitization and allergic diseases (Abrahamsson et al., 2011). When the bacterial populations are stabilized, and they image the adult’s gut microbiome composition, the intestinal immune system is considered to be ‘mature.’ Usually, this takes place around the second year of life (Delcenserie et al., 2008). In the gut-lung axis conceptualization, probiotic bacteria settled in the gut wield an influence on respiratory allergies and can relieve the symptoms. The data available are intriguing. It was observed that probiotic strains of *Lactobacillus acidophilus* NCFMTM (ATCC 700396) and *Bifidobacterium lactis* BI-04 (ATCC SD5219), which reside within the gut, have an impact on allergic rhinitis and can decrease the seasonal symptoms for birch and alder pollen allergies (Ouwehand et al., 2009), but the changes in the cytokine profile were not strongly pronounced in this case. *Lactobacillus* GG (LGG) and *Lactobacillus gasseri* TMC0356 (TMC0356) reduce seasonal symptoms of Japanese cedar pollen and inhibit the IL-4 and/or IL-5 production (Kawase et al., 2009). *L. acidophilus* strain L-92 can decrease ocular and nasal symptoms of perennial respiratory allergies (Ishida et al., 2005), but the Th1/Th2 changes were not observed. Those data suggest that the mechanism of probiotic alleviation of allergy symptoms may vary and more research is needed to understand the mutual interaction between the host and probiotic bacteria. The macroscopic changes (expressed by the clinical symptoms) always depend on the immunological state, and if probiotics indicate strong immunomodulatory properties. One of the modus operandi of probiotics is the influence of IgA secretion by B cells. Those immunoglobulin present on the mucus layer indicate a protective value and represent the first line of defense. IgA protects against viruses, neutralizes toxins and prevents pathological bacterial adhesion and their penetration through the epithelial barrier (Kotani et al., 2014). The low level of IgA-coated fecal bacteria in the first year of life seems to be linked to the development of allergy symptoms in a subsequent period (Dzidic et al., 2017). The mechanism of IgA production in contact with probiotic bacteria is still not fully clear. Kawashima et al. (2018) presented data which indicated that *Pediococcus acidilactici* K15 (one of the lactic acid bacteria, LAB) induced the production of this immunoglobulin via stimulation of dendritic cells, which produced IL-6 and IL-10. Moreover, the authors conducted a randomized, placebo-controlled trial using heat-killed K15 (oral supplementation) and indicated that those strains enhance the level of IgA also in the saliva of the patients, which confirmed a wide-operating range. In a respiratory allergy aspect, an IgA layer can trap an allergen before it reaches the epithelial barrier. In adult asthmatic patients, the severity of symptoms is directly related to a low level of IgA (Balzar et al., 2006; Kim et al., 2017; Ladjemi et al., 2018). These findings indicate that the microbiome’s consistency and composition are particularly important since it provides the first line of protection. Therefore, all activities leading to the maintenance or restoration of the microbiome may represent a new therapeutic approach. Recently it was shown that *Lactobacillus rhamnosus* GR-1 can alleviate the symptoms in the murine model of allergy (Spacova et al., 2020). In the study, mice were supplemented with 10^10^ CFU of bacteria four times per week through 6 weeks. Sensitization and asthma were induced by recombinant Bet v1 (rBet v1). *L. rhamnosus* GR-1 attenuated the hypersensitivity, reduced eosinophilic infiltration (assessed in bronchoalveolar lavage fluid, BALF) and protected against the impoverishment of the gut microbiota. Additionally, the authors made a recombinant modification of *L. rhamnosus*, which involved a vBet1- containing plasmid implantation into the bacterial cells. Except for the changes made also by a wild type of bacteria, the modified one additionally decreased the level of IL-1β and limited the presence of lymphocytes in the lungs (Spacova et al., 2020). The opportunity to use recombinant bacteria will be discussed later.

In another study, the properties of six species of *Lactobacillus* (*L. rhamnosus*, *Lactobacillus fermentum*, *Lactobacillus casei*, *Lactobacillus gasseri*, *Lactobacillus salivarius*, and *Lactobacillus reuteri*) and five strains of each species were checked (Li et al., 2020). Mice were intranasally challenged by house dust mite (HDM) extract. One week before and then during the whole process of sensitization, the mice were supplemented with probiotic strains (10^9^ CFU/strain/day). All the strains imposed a beneficial effect in an asthma-mimicking animal model. Nevertheless, the most promising strain seems to be *L. reuteri*, which limited the inflammation state expressed by cell infiltration to the lungs and histological scores, decreased the immunoglobulin level (IgE and IgG1) and cytokine level (IL-5 and IL-13). Additionally, *L. reuteri* indicated the influence on the gut microbiota by increasing butyrate production, which reduced inflammation in lungs and Th2 activity. However, it did not reduce the IL-17A. This effect was visible only for the *L. fermentum*, *L. casei*, and *L. gasseri*. Those data suggest that the *Lactobacillus* species have an impact on the immunity in the allergic model, but affect a different part of the response, Th2 and/or Th17. Therefore, the carefully selected strains can modulate the specific compartment of the allergic disease. The exact determination of the asthma type seems to be crucial for the targeted therapies. Additionally, this effect can be strengthened by additional prebiotic usage. In another study based on the murine model with HDM-induced asthma, oral administration of *L. rhamnosus* GG (LGG) alone (probiotic) (10^5^ or 10^7^ CFU/ml), and in a mixture with turmeric powder (symbiotic, 20 mg/kg mouse) indicated significant properties. A mixture of bacteria and probiotic reduced the airway hyper-responsiveness (measured by the methacholine response), inflammatory cell infiltration to the lungs, as well as decreased the Th2 and Th17 response, attenuated serum IgE, and increased the CD25^+^Foxp3^+^Treg population in splenocytes. A similar beneficial effect was observed after supplementation with turmeric powder alone (prebiotic), however, the mixture effect was stronger (Ghiamati Yazdi et al., 2020). Those data suggest that some of the food-derived products act synergistically with the selected bacteria, and as a mixture can improve the conventional therapy.

The valuable effect of probiotic in asthma was also confirmed in a study with ovalbumin (OVA) sensitization and challenge. Wu et al. (2016) induced asthma in the murine model. The mice received LGG powder (TTY Biopharm Company Limited, Taipei, Taiwan) as a probiotic drink containing *L. rhamnosus* GG, before or after the intraperitoneal OVA sensitization. The pre-treated group indicated a lower airway hyper-responsiveness in comparison with the post-treated group. Supplementation with *L. rhamnosus* reduced the serum level of IgE, IgG2a and cell infiltration to the lungs. The cytokine profile underwent significant changes as well. Probiotic supplementation was linked with an increase in Th1 cytokines profile (IL-12, IFN-γ, and TGF-β) and a decrease in Th2 cytokine response (IL-4, IL-5, IL-10, and IL-13).

It is worth noting that the beneficial effect of probiotics in respiratory allergy can largely depend on the model chosen. Casaro et al. (2018) indicated that the effectiveness of oral supplementation with *Bifidobacterium adolescentis* ATCC 15703 is different in Balb/c and C57BL/6 mice. In the experiment, both groups of mice were supplemented with 10^8^ CFU of bacteria 15 days before the first OVA sensitization, continuing until the last challenge (22nd day). In Balb/c mice, probiotic attenuated the eosinophil infiltration into the airway and increased IL-10 and IFN-γ local production (estimated in BALF). Those findings were not confirmed in a B6 group, which indicates that in the course of the allergy and its treatment, the genetic background can have a strong influence.

The promising effect of probiotic supplementation in allergy disease seems to be widely confirmed in an animal model. The studies with oral supply indicate the strong relationship between lung and gut as well as confirm the existence and importance of the gut-lung axis. The animal model is widely used in allergy studies. Studies conducted with probiotic and allergy are generally very promising. Nevertheless, they have limitations. The allergy/asthma induced by OVA, rBet, or HDM does not fully reflect the human allergy state, which is a multifactored disease. The animal model-based researches are mostly ‘clear’ benchmark, which is focused only on a selected aspect of allergy (does not include factors such as age, gender, hormones, resident microbiota, air pollution, dietary impact, additional epithelial barrier disturbances, accompanying diseases, drugs, and many others) (Spacova et al., 2018). For these reasons, the clinical trials with oral administration of probiotic in an allergy do not look so promising. One of the randomized, double-blind controlled trials run by Cabana et al. (2017), estimated the usefulness of *L. rhamnosus* GG (LGG) supplementation among infants in high-risk groups, among others for asthma and rhinitis. For the first 6 months of life, infants were supplemented by 10^9^ CFU of *L. rhamnosus* GG (LGG) and prebiotic (inulin). The control group received inulin alone. Results do not confirm the beneficial effect. *L. rhamnosus* did not prevent asthma in comparison with the control group. At 2 years of age, 30.9% of children developed eczema in the control arm and 28.7% in the probiotic arm. At 5 years of age, the incidence of asthma was 17.4% in the control and 9.7% in the LGG arm (without statistical significance) and the risk of asthma was higher for children with eczema. Nevertheless, supplementation took place only among infants and within the first 6 months. Additional prenatal supplementation during pregnancy and/or longer duration of the treatment might be more effective (Cabana et al., 2017). Another randomized, placebo-controlled trial was based on an oral supplementation with *L. reuteri* ATCC 55730 (10^8^ CFU). Families (*n* = 232) with allergy history were included in the study. The supplementation process started in the last month of pregnancy and was continued through the first year of the child’s life. The long-term effect was assessed after 7 years (based on clinical assessment, spirometry, exhaled nitric oxide and skin prick tests). 184 children completed a follow-up study (Abrahamsson et al., 2013). There were no significant changes observed between the probiotic and placebo group in the prevalence of asthma or allergic rhino-conjunctivitis (asthma: 15% in a probiotic group vs. 16% in placebo; rhino-conjunctivitis: 27% vs. 20%). However, the study has some limitation, also pointed out by the authors. According to the available literature, the beneficial effect of probiotics is obtained by long-term usage. In the high-risk group of children, early supplementation, even in the second trimester of pregnancy can be more effective. Recent research showed that the fetus’s intestine is colonized by bacteria, including *Lactobacillus* (Rackaityte et al., 2020). Additionally, the fetus is exposed to bacteria as well as allergens, which can modulate its immune system (Rackaityte and Halkias, 2020). The early fetus exposition for probiotic strains can be beneficial both for the early colonization of the intestine as well as for the early challenge of T cells. The results obtained by Abrahamsson et al. (2013) can be linked also to the dose of probiotic.

Nevertheless, a meta-analysis of randomized controlled trials conducted by Wei et al. (2020), evaluated the previous data of probiotic usefulness with regard to the infants’ protection against asthma incidences. In the analysis, 19 reports published 2003–2018 were included. The authors do not confirm the dependence between probiotics and asthma. However, the link between probiotics and allergy was found, which was expressed by a reduction of wheeze incidence among infants with atopic disease.

The above data suggest that the beneficial effect of probiotics in the first years of life can be insufficient, but in later life could be promising. Del Giudice et al. (2017) published data where the usefulness of *Bifidobacterium mixtures* [*Bifidobacterium longum* BB536 (3 × 10^9^ CFU), *Bifidobacterium infantis* M-63 (1 × 10^9^ CFU), and *Bifidobacterium breve* M-16 V (1 × 10^9^ CFU)] was determined. Forty children in mean age 9 years old were supplemented with one sachet (3 g) each of probiotic mixture per day for 8 weeks. The group of supplemented children showed attenuation of symptoms and improved quality of life (based on Mini Rhino conjunctivitis Quality of Life Questionnaire). This finding agrees with another report, where the changes in the fecal microbiota in adult patients with long term asthma (mean age 39.43 ± 10.98 years old) were noted (Hevia et al., 2016). Those patients had decreased *Bifidobacterium* population, so supplementation with those strains could be a novel therapeutic target. Additionally, it was observed, that among the *Bifidobacterium* population, the *B. adolescentis* prevailed, which is not common among healthy individuals.

Other research indicated the usefulness of the *Lactobacillus* strains in a group of asthmatic children in the age 6–18 years (Huang et al., 2018). A double-blind, randomized, placebo-controlled trial included 153 patients, who were supplemented with *Lactobacillus paracasei* GMNL-133 (BCRC910520 and CCTCC M2011331), *L. fermentum* GM-090 (BCRC 910259 and CCTCC M204055), their mixture or a placebo. The supplementation was continued for 4 months. In all probiotic groups, the Childhood Asthma Control Test and asthma severity decreased in comparison with the placebo group. Differences among the probiotic groups were not observed. The total level of serum IgE decreased only in the group with mixtures of *L. paracasei* and *L. fermentum*. The changes in IFN-γ, IL-4, and TNF-α were not observed. Those results suggest that the mixture of some strains of probiotics have a stronger impact on the allergy disease in comparison with the single strain. Those observations were confirmed also in an aspect of different diseases, such as necrotizing enterocolitis (Chang et al., 2017), colitis, allergic diarrhea (Atarashi et al., 2013) and others. Those phenomena can be linked to the amount of bacteria received by patients and/or the synergistic action of bacteria.

A summary of the examples of the effect of oral administration of probiotics on respiratory allergy is contained in Table 1.

**TABLE 1:** The effect of oral administration of probiotics on respiratory allergy.

Probiotic strain	Model of study	General effect	Source
**Animal model**			
*Lactobacillus rhamnosus* GR-1 recombinant *Lactobacillus rhamnosus* (containing plasmid with *vBet*1)	Murine model	↓Hypersensitivity symptoms, ↓Eosinophilic infiltration, Protection against the impoverishment the gut microbiota. ↓IL-1β. ↓Lymphocytes population in lungs.	Spacova et al., 2020
*Lactobacillus* (*L*. *rhamnosus*, *L*. *fermentum*, *L*. *casei*, *L*. *gasseri*, *L*. *salivarius*, and *L*. *reuteri*) *L*. *reuteri*	Murine model (HDM)	All strains indicated some beneficial effect. ↓Cell infiltration to the lungs, ↓Histological scores. ↓IgE, ↓IgG1, ↓IL-5, IL-13, ↑Butyrate production, ↓Th2 activity.	Li et al., 2020
*Lactobacillus rhamnosus* GG (LGG) alone, and in mixture with turmeric	Murine Model (HDM)	↓Airway hyperresponsiveness ↓Inflammatory cells infiltration to lungs, ↓Th2 cytokine profile, ↓serum IgE.	Ghiamati Yazdi et al., 2020
*Lactobacillus rhamnosus* GG	Murine model (OVA)	↓IgE, ↓IgG2a, ↓cell infiltration to the lungs, ↑Th1 cytokines profile (IL-12, IFN-γ, and TGF-β), ↓Th2 cytokine response (IL-4, IL-5, IL-10, and IL-13)	Wu et al., 2016
*Bifidobacterium adolescentis* ATCC 15703	Murine model Balb/c and C57BL/6 (OVA)	Balb/c mice: ↓eosinophil infiltration into airway, ↑IL-10, ↑IFN-γ local production (estimated in BALF), C57BL/6: no effect	Casaro et al., 2018
**Clinical trials**			
*Pediococcus acidilactici* K15	Healthy volunteers	↑IgA, activation B cells through IL-6 and IL-10 produced by dendritic cells	Kawashima et al., 2018
*Lactobacillus rhamnosus* GG (LGG) and inulin (prebiotic)	A randomized double-blind controlled trial, infants	No preventive effect against asthma in comparison with the control group	Cabana et al., 2017
*L. reuteri* ATCC 55730	A randomized placebo-controlled trial, fetus (last months of pregnancy) and infants	No effect assessed after 7 years	Abrahamsson et al., 2013
*L. rhamnosus, L. reuteri, Lparacaseii, L. acidophilus, B.breve*	Meta-analysis of randomized controlled trials	No dependence between probiotic and asthma,↓wheeze incidence among infants with atopic disease.	Wei et al., 2020
*Bifidobacterium* mixtures (*B. longum* BB536, *B. infantis* M-63, *B. breve* M-16 V)	Children (mean age 9 years old)	↓Symptoms, ↑quality of life	Del Giudice et al., 2017
*Lactobacillus paracasei* GMNL-133 (BCRC910520 and CCTCC M2011331), *Lactobacillus fermentum* GM-090 (BCRC 910259 and CCTCC M204055). Mixture of *Lactobacillus paracasei* and *Lactobacillus fermentum*	A double-blind, randomized, placebo-controlled trial, children (6–18 years old)	↓Asthma severity, ↓scores of the Childhood Asthma Control Test, ↓total IgE	Huang et al., 2018
*Lactobacillus acidophilus* NCFM^TM^ (ATCC 700396) and *Bifidobacterium lactis* BI-04 (ATCC SD5219),	Children with birch pollen allergy	↓Infiltration of eosinophils in the nasal mucosa, ↓seasonal symptoms, ↑IL-10	Ouwehand et al., 2009
*Lactobacillus GG (LGG)* and *Lactobacillus. gasseri TMC0356 (TMC0356)* in a fermented milk	Double-blind placebo-controlled clinical study	LGG:↓IL-4 and IL-5, TMC0356: ↓IL-5	Kawase et al., 2009
*Lactobacillus acidophilus* L-92	Randomized, double-blind, placebo-controlled clinical trial	↓Nasal and ocular symptoms,	Ishida et al., 2005

Generally, oral supplementation of probiotic strains seems to be promising, nevertheless, it still has many moot points. The longevity of probiotic usage seems to be important. The data indicate that a beneficial impression relates to long-term therapy. A beneficial effect on the infants in an allergy risk group can be obtained when supplementation occurs *in utero*, when certain tolerance mechanisms are formed. However, reports showing the fetal gut’s microbiome are very new and need further consideration.

Another issue is the model chosen. The available reports are based on different models which could be the reason for the contrary results. The most popular animal model of asthma is a murine model. However, as indicated in the study run by Casaro et al. (2018), genetic background is one of the most important factors determining the probiotic effect. The genetic background in human cannot be unified, as it takes place in murine models. In a study based on a human model, additional factors such as age, gender, previous treatment, diseases, and others seem to be particularly important. These elements cannot be mimicked in cellular or animal models. Another issue is an applied procedure and allergy-trigger factor. For instance, mice do not develop asthma naturally, so the inflammatory state within the respiratory tract must be induced by sensitization and re-exposure to the chosen factor. In contrast to human asthma, the long-lasting repeatable provocation with the antigens leads to tolerance. In humans, sensitization takes place via the respiratory tract, whereas in some study, e.g., OVA (which generally is not considered as an asthma-inductor in humans) is administered via intraperitoneal injection (Chapman et al., 2014; Mullane and Williams, 2014; Aun et al., 2017). Additionally, the allergic disease can overlap, and have specific endotypes and phenotypes. Therefore, the animal models, although they are widely used, never fully reflect a human individual’s asthma, and the effect visible in an animal-based study can be differently expressed in clinical trials.

It is also interesting that some of the studies report an improvement in the severity of the symptoms, but do not show a significant change in the cytokine profile (Ishida et al., 2005; Ouwehand et al., 2009). This lack of statistical differences can be an effect of a small study group. Nevertheless, it can be pointed out that other, still unknown, processes can have a significant influence on the pathogenesis of allergies. Previously asthma was thought to be an allergic disease. Today it is known that there are many types of asthma with different mechanisms. The impact of the microbiota on asthma is indicated, nevertheless, the exact path is still blurred. The probiotics vary in their modulatory properties. The supplementation with carefully selected strain and the additional strengthening by the prebiotic can be a novel trend in the support of conventional therapy. The above presented probiotic usefulness in allergy diseases is based on the gut-lung axis. The gut microbiome composition depends on many factors, such as mode of birth, diet, lifestyle, geographic location, usage of antibiotics and others (Ver Heul et al., 2019). The altered gut microbiota in early childhood correlates with atopic disease development (Kozik and Huang, 2019; Ver Heul et al., 2019). The bacterial populations compete with each other for space and nutrients and can modulate or battle each other. Therefore, there is a chance that the supplemented probiotic will not anchor into its specific niche. Only a thorough analysis of the composition of the patient’s gut microbiome would allow supplementing or remediation of the appropriate bacterial populations, typical for healthy individuals. The condition of the intestinal microbiome seems to be decisive. Impoverished, hostile, or altered intestinal flora can attenuate the beneficial effect of probiotics. Therefore, recent years of investigations showed that an intranasal supply of probiotic can be very promising.

## Effect of Intranasal Administration of Probiotics on Respiratory Allergy

The function of the gut-lung axis is possible due to the existence of their common immune system and the available data showed that the influence of both organs is equivalent, the intestinal microbiota affects the respiratory system, and the respiratory microbiota actively affects the intestines (Budden et al., 2016; Dumas et al., 2018; Zhang et al., 2020). It should be noted that oral probiotic formulations must pass through the digestive system where they are subjected to the action of various digestive juices. It could have an impact on the functionality of the probiotic strains and be linked with a desired long-term therapy. An altered microbiota of the respiratory system influences the pathogenesis of the respiratory allergy (Remot et al., 2017), therefore the nasal administration of probiotics can be greatly beneficial. Unlike oral supplementation, this could work directly within the affected tissues, which should bring a faster and stronger effect. The intranasal administration of the probiotic strains was determined extensively in various models. The beneficial effect was confirmed in a murine model of respiratory viral infection for *Lactobacillus* (Choi et al., 2015; Kumova et al., 2019), on the immune cells in nasal mucosa and tonsils in a piglet model for *Bacillus subtilis* (Yang et al., 2018) and as a prevention against acute otitis for *S. salivarius* 24SMB (Marchisio et al., 2015). However, such reports about the intranasal administration of probiotic in the respiratory allergy are in the minority. It should be underlined that each niche of the microbiome residency differs from the other in the composition of its bacterial population. Therefore, the kind of probiotic strain should be carefully chosen. In a murine model of asthma, induced by birch pollen, a different mode of transient colonization was indicated (Spacova et al., 2019). Mice were intranasally supplemented with *Lactobacillus rhamnosus* GG and *L. rhamnosus* GR-1 in dose 5 × 10^8^ CFU (8 times per day, 2 weeks before asthma induction). Live bacteria were detectable in the nose and gut in 24 h of observation, not longer. There were significant differences in the size of bacterial populations: in the nose *L. rhamnosus* GG cells (5.2 × 10^4^–1.7 × 10^5^ CFU/mouse) and *L. rhamnosus* GR-1 (1.6 × 10^3^–10^4^ CFU/mouse), in fecal samples: *L. rhamnosus* GG cells (1.9 × 10^4^–8 × 10^5^ CFU/g feces) and *L. rhamnosus* GR-1 (0–2.4 × 10^4^ CFU/g feces), in the cervical lymph nodes *L. rhamnosus* GG (only) (6 × 10^1^–3.6 × 10^2^ CFU/mouse). In a modulated bacterial population (expressing a fluorescent dye, mCherry), the adhesive properties to the nasal mucosa cells were visible only for *L. rhamnosus* GG. This strain also more strongly decreased the Bet v1 specific IgG1, eosinophil infiltration (visible in BALF and lung histology) and IL-5, IL-13 level in the lung. *L. rhamnosus* GG attenuated the airway hyperreactivity, which was less pronounced for *L. rhamnosus* GR-1. For both, *L. rhamnosus* GG and *L. rhamnosus* GR-1, no changes were observed for IgG2, IgE and cytokine profile (IL-4, IL-10, IL-17, and IFN-γ), as well as in Gata3, Tbx21, Rorc, or Foxp3 mRNA (Spacova et al., 2019). Those data underline that the different strains of the same species can participate in immunological response in a different way, and the improvement in symptoms (expressed as airway hyperreactivity) is not necessarily reflected in a typical cytokine profile. The adhesive properties of *L. rhamnosus* GG can cause a better general ‘outcome’. As was proven within the gut epithelial barrier, probiotic bacteria improved the unsealed tight junctions and participated in wound repair (Hsieh et al., 2015). IL-5 produced by bronchial epithelial cells is linked to eosinophils infiltration and IL-13 seems to induce a loss of the functions of the epithelial respiratory epithelial cells including ciliated cells (Wu et al., 2010; Seibold, 2018; Barretto et al., 2020). Therefore, decreasing those cytokines can be beneficial and attenuate the symptoms.

Another important cytokine linked with a respiratory allergy is IL-10. It plays a crucial role in Th2 response. It can control Th2 survival and regulate allergy inflammation insensitivity (Coomes et al., 2017). The intranasal administration of *Clostridium butyricum* extract in the murine model of allergy rhinitis resulted in an increased level of IL-10 produced by epithelial cells (Zeng et al., 2019). Additionally, epithelial cells incubated with bacterial extract affected B cells by increasing IL-10 expression. That subpopulation of B cells has a significant impact on immunosuppression and inhibits experimental allergy rhinitis. The data presented underline the magnitude of the respiratory epithelial cells in an allergy pathomechanism, as well as the regulatory role of probiotic bacteria.

In another study, the immunomodulatory properties of *L. paracasei* NCC2461 and *Lactobacillus plantarum* NCC1107 were determined (both 10^9^ CFU) (Pellaton et al., 2012). In the murine model, the allergy inflammation was conducted by OVA. Bacteria were administrated intranasally (*L. paracasei* NCC2461 and *L. plantarum* NCC1107) or intragastrically (*L. paracasei* NCC2461). The authors observed a protective impact of *L. paracasei* NCC2461 in intragastrical administration during the OVA aerosol exposure expressed by a lower cell requirement in BALF. This effect was not observed during the sensitization phase. Intranasal administration of *L. paracasei* NCC2461 reduced the numbers of eosinophils in BALF, decreased the level of IL-5 and eotaxin production in the lungs, and increased the Treg population in the respiratory tract. In comparison with and *L. plantarum* NCC1107, changes induced by *L. paracasei* NCC2461 in internasal administration were more pronounced. The general result suggests that *L. paracasei* NCC2461 can be effective during the acute phase of the allergy, such as during plant dusting. However, the level of specific IgE was not affected, which can be the effect of the too-short supplementation (Pellaton et al., 2012). These results underline, that probiotic strains can act differently, therefore the selection of appropriate strains seems to be crucial. Another study, based on the murine model and poly-sensitization by birch and grass main allergens, confirmed the beneficial effect of *L. paracasei* NCC2461, as well as *B. longum* NCC 3001 after intranasal administration (Schabussova et al., 2011). Mice were sensitized by the recombinant allergens (rBet v1, rPhl p1, and rPhl p5) and then challenged by the native birch and grass pollens. Application of probiotic bacteria took place at the time of sensitization and challenge (experiment 1: 5 × 10^8^ CFU bacteria supplemented 4 h before each poly-sensitization and 4 h before each challenge), or before sensitization (experiment 2: 10^9^ CFU bacteria supplemented during days 0–3, and then 5 × 10^8^ CFU, days 7–10). At the time of sensitization *L. paracasei* significantly reduced the IgE-dependent basophil degranulation against Bet v1 and *B. longum* NCC against Phl p5. The changes in the allergen-specific IgG antibody in serum were observed neither in the Th2-related IgG1 nor in the Th1-related IgG2a. A reduction of IL-5 and IFN-γ in the spleen was observed. Nevertheless, a different mode was indicated. *B. longum* changed the level of IL-5 to Bet v1, Phl p1, and Phl p5 and IFN-γ to Bet v1, Phl p1. Both strains reduced the eosinophils infiltration in BALF. Changes were not visible for numbers of neutrophils and macrophages. The increase of Bet v 1-specific IgA in BALF was observed in both strains. In contrast, during probiotic administration prior to sensitization and challenge, only *B. longum* NCC 3001 reduced eosinophil numbers and strongly reduced the level of IL-5 in BALF. Additionally, in the spleen and lung, the change in IL-10 level was not observed, but for both strains, IL-10 mRNA measured in submandibular and bronchial lymph nodes indicated its increase. The authors suggest that both probiotic bacteria can attenuate Th1 and Th2 response when the administration takes place at the time of sensitization and challenge. Additionally, *B. longum* administrated before poly-sensitization can attenuate the Th2 response. Results obtained show that firstly different strains have different features and can participate in an allergy attenuation via distinct ways, and secondly, intranasal administration seems to have strong local activity. An interesting scientific trend is research-based on recombinant bacteria. The allergen molecules expressed intra- or extra-cellularly, strengthen the effect of probiotic and seems to be a new therapeutic approach (Cano-Garrido et al., 2015). Sarate et al. (2019) conducted the experiment where recombinant probiotic was used. In the murine model, the poly-sensitization was induced by rBet v1, rPhl p1, and rPhl p5 (birch and grass main allergens). Modified bacteria (expressing those allergens) were administrated intranasally and, for comparison purposes, also by oral dose. Recombinant bacteria *Escherichia coli* Nissle 1917 decreased the level of Th2 related cytokine IL-5 and IL-13 in BALF, reduced cell infiltration and mucus production in the lung. Moreover, the allergen- specific IgE antibody levels against Bet v1, Phl p1, and Phl p5 in serum were reduced, and the IgA level was increased. The upregulation of Foxp3, TGF-β, and IL-10 mRNA in the bronchial lymph nodes was noted as well. The oral supplementation did not affect the specific parameters above. Additionally, the authors tracked the fate of *E. coli* Nissle 1917 after oral and intranasal administration. In both cases, the bacteria did not permanently colonize the nose/lung/gut. These data suggest that the internasal administration of probiotic with allergen expressing properties has a strong impact on the course of the allergy. This could have an effect on the activity *in situ* in the respiratory tract. The route of oral administration and its lung reflection is much longer. Therefore, the changes may not be visible with the short-term use of probiotics. Since recombinant *E. coli* Nissle 1917 does not colonize the respiratory and gastrointestinal tract, it can make a good vector for allergens, especially in the context of allergen desensitization. Nevertheless, this approach needs further investigation. The beneficial effect of recombinant strains expressing allergens has also been described elsewhere. Schwarzer et al. (2011) conducted a study based on the murine model where germ-free mice were colonized by recombinant *L. plantarum* (expressing rBet v1). Then the neonatally mono-colonized mice (off-spring monocolonized with recombinant bacteria via their mother) were subjected to sensitization by rBet v1. In an *in vitro* experiment, splenocytes from monocolonized naive mice stimulated by rBet v1 showed an increased production of IFN-γ and undetectable levels of IL-4 and IL-5, which indicated induction of the Th1 response. The antibodies’ level (IgG1, IgG2a, IgA, or IgE) was not altered. In contrast, in neonatally mono-colonized mice sensitized with the allergen, the level of IgG1, IgG2a, total and specific IgE was reduced. There were no changes observed in IgA in serum. In spleen cell cultures, the level of IL-4 and IL-5 was suppressed, and IFN-γ increased. In the mesenteric lymph nodes, similar changes for IL-5 and IFN-γ were observed. Some changes were also observed in the level of Foxp3 mRNA, IL-10, IL-10 mRNA and TGF-β. Nevertheless, except for Foxp3, they do not reach statistical significance. These findings indicate that recombinant bacteria can induce a Th1 response. Differences in the two experiments (with cell culture from naïve colonized mice stimulated with allergen vs. colonized mice sensitized with an allergen) suggest that there are other players in the allergy pathomechanism and probiotic action. Nevertheless, the above findings are in concordance with other studies, where the intranasal administration reduced the cytokine level and upregulated the regulatory mRNA (Foxp3). Table 2 summarizes the effects of intranasal administrated probiotics on respiratory allergy.

**TABLE 2:** The effect of intranasal administrated probiotics on respiratory allergy.

Probiotic strain	Model of study	General effect	Source
*Lactobacillus rhamnosus* GG and *Lactobacillus rhamnosus* GR-1	Murine model (native birch pollen-induced asthma)	*L. rhamnosus* GG: ↓ Bet v specific IgG1, ↓eosinophil infiltration, ↓IL-5, IL-13 in lung, ↓ the airway hyperreactivity.	Spacova et al., 2019
*Clostridium butyricum* extract	Murine model of allergy rhinitis (OVA)	↑IL-10 produced by epithelial cells, and B cell	Zeng et al., 2019
*Lactobacillus paracasei* NCC2461 and *Lactobacillus plantarum* NCC1107	Murine model of allergy, (OVA)	*L. paracasei:*↓ eosinophils, ↓IL-5, ↓ eotaxin production in the lung, ↑Treg population in the respiratory tract not so strong effect of *L.plantarum* NCC1107	Pellaton et al., 2012
*Bifidobacterium longum* NCC 3001 and *Lactobacillus paracasei* NCC 2461	Murine model (poly-sensitized with rBet v1, rPhl p1 and rPhl p5)	↓IL-5, ↓IFN-γ, ↓eosinophile infiltration. ↑IL-10 mRNA in submandibular and bronchial lymph nodes, ↑IgA for rBet v1,	Schabussova et al., 2011
Recombinant *Escherichia coli* Nissle 1917	Murine model, poly-sensitization induced by rBet v1, rPhl p1, and rPhl p5	↓IL-5,↓ IL-13 in BAL, ↓ cells infiltration and mucus production in the lung, ↓ IgE against Bet v1, Phl p1, and Phl p 5 in serum, ↑IgA level, ↑Foxp3, TGFβ, and IL-10 mRNA in bronchial lymph nodes	Sarate et al., 2019
Recombinant *Lactobacillus plantarum* NCIMB8826	Murine model of allergy, (rBet v1)	↓IgG1, IgG2a, total and specific IgE, ↓ IL-4, ↓IL-5, ↑Foxp3 mRNA	Schwarzer et al., 2011
			

Intranasal administration of probiotics seems to be at the cutting edge of new research direction. In respiratory disorder supplementation by nose might be more effective, because (1) it avoids the gastrointestinal tract, so the risk of bacteria damage is decreased, (2) probiotic works in its target place, having a stronger effect on the local immunity and (3) recombinant bacteria might help in allergen tolerance acquisition, which could be a background to a promising novel therapy. However, there are some limitations. Similar to oral supplementation, the study model seems to be very important, especially in the aspect of respiratory tract structure and properties. Apart from the nose’s anatomical differences, in contrast to humans, mouse NALT is placed in a certain site, and antigen uptake is done by dendritic cells and M cells independently (Bienenstock and McDermott, 2005; Pabst, 2015). BALT is only occasionally presented in mice and is induced by stimuli such as infection or inflammation, whereas in humans it is continuously expressed during childhood and early adulthood (Heier et al., 2011). These anatomical and functional differences might have an impact on biomedical research. The doses of probiotics must be considered as well. Most of the available data is based on similar amounts of supplemented bacteria, by both the oral and nasal route. Increasing the doses of probiotics used (especially oral ones) could get a stronger effect. Nevertheless, it should be remembered that a ‘healthy’ microbiome contains different types of bacteria in a certain balance. Therefore, a targeted therapy should begin with the identification of the bacterial composition. Carefully selected probiotics may affect specific compartments of the immune response and constitute a supplement to individual therapy.

## Conclusion

**FIGURE 2:**
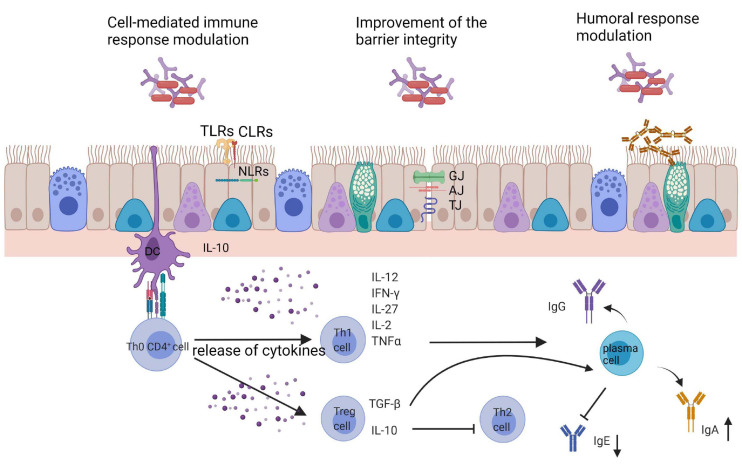
General directions of the action of bacterial cells on the host. GJ, gap junction; AJ, adherence junctions; TJ, tight junctions; TLRs, toll like receptors; CLRs, C-lectin receptors; NLRs, NOD-like receptors; DC, dendritic cells.

To summarize, probiotics seem to be promising in the process of immunomodulation in the course of respiratory allergy. Figure 2 shows the general directions of the action of bacterial cells on the host. Both oral and intranasal supplementation indicate the beneficial effect in a limitation of the inflammatory processes. The impact of probiotic in a respiratory allergy is pronounced by:

(1) Changes in Th1/Th2 skewing: increase in Th1 cytokines profile (IL-12, IFN-γ, and TGF-β), decrease in Th2 cytokine profile (IL-4, IL-5, IL-10, and IL-13),
(2) Decrease in eosinophil and lymphocytes infiltration to the respiratory tract and IgE, IgG1, and IgG2a production,
(3) Increase in butyrate and IgA production,
(4) Symptoms alleviation and improvement in quality of life.

Some of the discrepancies between the animal model and clinical trials can be a result of differences in the applied models. Animal-based studies are narrowed to specific conditions. The clinical trials include many variables such as accompanying disease, age, gender, and many other factors. Intranasal administration of probiotic in a respiratory allergy seems to be promising. Additionally, the usage of recombinant bacteria can be a novel allergen-based therapy in the future. Probiotics as vectors not only can improve clinical tolerance for the allergen but can also improve the state of the epithelial barrier, the first line of defense against foreign particles. Extensive studies are needed to confirm the beneficial effect as well as the safety of this approach.

## Author Contributions

DJ: manuscript preparation and review corrections. SG: manuscript correction and figures drawing. Both authors contributed to the article and approved the submitted version.

## Conflict of Interest

The authors declare that the research was conducted in the absence of any commercial or financial relationships that could be construed as a potential conflict of interest.

## Footnotes

1 World Health Organization, https://www.who.int/respiratory/asthma/definition/en/ (accessed 1.05.2021).

2 National Institutes of Health (NIH) (2015). The Lung HIV Microbiome Project (LHMP) https://biolincc.nhlbi.nih.gov/studies/lhmp/ (accessed 28.02.2021).
